# Regular Review of Anticholinergic Burden to Reduce Falls Risk in Elderly Inpatients

**DOI:** 10.1192/bjo.2026.11382

**Published:** 2026-06-30

**Authors:** Prabin Gautam, Samrana Khan, Wan-Ting Yew

**Affiliations:** Kent and Medway Mental Health NHS Trust, Dartford, United Kingdom

## Abstract

**Aims:**

Elderly patients in psychiatric settings are particularly vulnerable to falls, a risk exacerbated by the side effects of psychotropic medications. The cumulative effect of thesedrugs, known as the Anticholinergic Cognitive Burden (ACB), can cause dizziness, confusion, and impaired balance. Prior to this project, ACB scores were not routinely calculated or used to guide medication reviews on our older adult inpatient ward.

The primary aim was to reduce the risk of falls by implementing routine monitoring of ACB scores and ensuring weekly medication reviews. The hypothesis was that increasing awareness of a patient's anticholinergic load would lead to proactive deprescribing and a reduction in the average ACB score by the time of discharge.

**Methods:**

We conducted a Quality Improvement Project on Jasmine Ward, an inpatient ward for elder females. Data was collected retrospectively from electronic patient records. Cycle 1 (n=37) established baseline practice. Cycle 2 (n=36) involved the introduction of a protocol requiring weekly multidisciplinary reviews for patients with an ACB score >3. The intervention focused on prompting clinicians to consider deprescribing high-burden medications.

**Results:**

The documentation of medication reviews improved from 54.5% in Cycle 1 to 83.3% in Cycle 2. Similarly, documented consideration of deprescribing increased from 39.3% to 50.0%.

Most notably, the intervention reversed the trend of accumulating drug burden. In Cycle 1, the average ACB score increased during the hospital stay (Admission: 3.17 to Discharge: 3.30). In Cycle 2, the average ACB score decreased during the stay (Admission: 3.46 to Discharge: 3.22). The incidence of falls remained comparable between cycles (21.6% vs 25.0%), suggesting that while drug burden was successfully reduced, the impact on fall rates may be a lagging indicator or influenced by the small sample size.

**Conclusion:**

Systematic monitoring of ACB scores is an effective strategy to drive deprescribing and reduce the pharmaceutical burden on elderly psychiatric inpatients. While the reduction in ACB scores did not immediately correlate with a drop in fall rates within the short study period, the improved documentation and the successful reduction of anticholinergic burden represent a crucial step towards improving long-term patient safety and cognitive health.

